# Characterization of the volatile components in the processing of *Citri Exocarpium Rubrum* black tea based on HS‐SPME and GC/MS

**DOI:** 10.1002/fsn3.4374

**Published:** 2024-08-15

**Authors:** Qiaoyi Zhou, Dongxia Liang, Caijin Ling, Liyang Gao, Zhi Ling

**Affiliations:** ^1^ Guangdong Provincial Key Laboratory of Tea Plant Resources Innovation and Utilization Tea Research Institute, Guangdong Academy of Agricultural Sciences Guangzhou Guangdong China; ^2^ Pingyuan Yuanshan Lake Agricultural Development Co., Ltd. Meizhou Guangdong China

**Keywords:** black tea, *Citri Exocarpium Rubrum*, volatile components

## Abstract

*Citri Exocarpium Rubrum* black tea (CER tea) is a novel blend tea infusion combining tea and CER. Due to its potential health benefits and unique flavor, it has gained popularity among consumers. Nevertheless, the aroma characteristics and key odorants of it remain to be distinguished and cognized. The HS‐SPME and GC/MS techniques were employed to analyze the alterations in volatile constituents throughout the critical stages of CER tea production. A total of 200 chemical compounds exhibited notable disparities during the processing procedures. Among them, terpenes and esters were the most abundant compounds in CER tea, which might be the key material basis for the aroma quality of CER tea. It is worth noting that 124 metabolites were significantly increased in the kneading stage and drying stage, including benzeneacet aldehyde, trans‐nerolidol, and D‐limonene, with contained floral and fruity odors, which might be closely related to the aroma of CER tea. Monoterpenes might be important contributors to the aroma of CER tea. This study provided a theoretical basis for the quality improvement of CER tea.

## Graphical abstract

The changes in volatile components during the key processes of *Citri Exocarpium Rubrum* (CER) black tea were analyzed by HS‐SPME and GC/MS. Among them, terpenes and esters were the most abundant compounds in CER tea, which might be the key material basis for the aroma quality of CER tea. It is worth noting that 124 metabolites were significantly increased in the kneading stage and drying stage, including benzeneacet aldehyde, trans‐nerolidol, trans β‐ionone, and β‐damascene, with contained floral and fruity odors, which might be closely related to the aroma of CER tea.

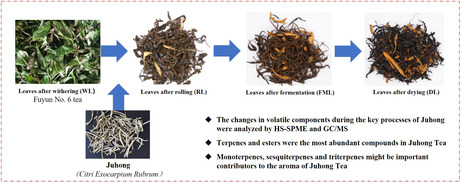

## 1 INTRODUCTION

Tea is a popular drink derived from various camellia species, and it holds the title for being the second most consumed beverage globally, following water. Among the different types of tea, black tea takes the lead in terms of both production and consumption, constituting approximately 75% of the overall tea market globally (Zhang et al., 2019). Due to its unique sensory characteristics (such as aroma, taste, etc.) and health benefits, it is widely popular worldwide (Wang, Jin, et al., 2019; Wu et al., 2019). Black tea refers to a type of tea that undergoes a complete fermentation process. The fermentation stage is considered crucial in the production of black tea and significantly impacts its overall quality. The enzymatic action of polyphenols, particularly catechin, in tea is predominantly controlled by the biochemical reaction of polyphenol oxidase and peroxidase. Simultaneously, by subjecting volatile compounds to a range of alterations, the scent undergoes a transformation, shifting from a grassy taste to a delightful and aromatic floral essence (Tan et al., 2016). Aroma is a key quality index of tea (Feng et al., 2019). In the realm of scientific investigation, a staggering number of 700 diverse aromatic compounds have been identified within the tea plant. Among these, approximately 80 can be found in fresh tea, while an impressive 400‐plus compounds have been discovered in black tea (Peluso & Serafini, 2017). The volatile compounds in black tea are mainly composed of alcohols, aldehydes, esters, hydrocarbons and ketones (Kang et al., 2019), among which linalool, linalool oxide, phenylethanol, phenylacetaldehyde, geraniol, (E)‐2‐octanal, 1‐octene ‐3‐ol, methyl salicylate, and (Z)‐jasmonate have been identified as the key aroma components of black tea (Alasalvar et al., 2012; Kang et al., 2019; Xiao et al., 2017).

*Citri Exocarpium Rubrum* (CER), also called “Juhong” in China, refers to the dried outer pericarp of *Citrus reticulata Blanco* and its cultivars. Renowned for its ability to alleviate respiratory ailments, dampness, and phlegm, this herbal remedy is predominantly cultivated and gathered in Zhejiang and Guangdong provinces (Con, 2020). It is rich in nutritive active ingredients, such as flavonoids, volatile oils, polysaccharides, and coumarins, which have high utilization value in the fields of medicine, food, and daily chemical industry. The Ministry of Public Health simultaneously published medicine and food homology. Additionally, Juhong is a renowned traditional Chinese medicine (Zhao et al., 2014).

CER tea is a specific Chinese blend tea product that adds “Juhong” in the black tea creation process after rolling, fermenting, and drying steps of finished products. It has the characteristics of CER and tea, mellow taste, and tangerine aroma and is widely favored by consumers. Previously, our research team had successfully produced a kind of CER tea. It was popular for consumers in the market because of its unique aroma. But there was currently a problem in flavor that the two flavors, tea and CER have not fused well. Therefore, how to improve the fusion degree of CER tea and further improve the quality of CER tea are urgent needs. Exploring the processing process of CER tea and further refining the process parameters were the key points to solve the above problems.

Volatile compounds are greatly influenced by various processing steps (Zheng et al., 2016), yet the absence of comprehensive elucidations regarding the fragrance characteristics of CER tea utilizing distinct processing techniques is evident. Analyzing the fragrance constituents of CER tea throughout its processing holds immense importance in understanding the fundamental elements contributing to its flavor and biological attributes. It also aids in uncovering the impact of processing techniques on the chemical constituents that determine its quality, thereby providing valuable insights for enhancing processing methods.

Effective extraction from tea matrix is crucial for precise qualitative and quantitative analysis of tea volatiles. Various extraction and preconcentration methods have been developed for this purpose, with separation and identification typically carried out using one‐dimensional or two‐dimensional gas chromatography and mass spectrometry (GC–MS, GC–GC/MS). Techniques such as liquid–liquid extraction, simultaneous distillation, solid‐phase extraction, solid‐phase microextraction, supercritical fluid extraction, and stirring rod adsorption extraction have all been extensively documented for the analysis of volatile compounds in tea (Foudil‐Cherif & Yassaa, 2012; Yang et al., 2016). Among these methods, headspace solid‐phase microextraction (HSPME) and GC–MS methods are extensively acknowledged techniques for the identification of volatile compounds in beverages and food items (Panighel & Flamini, 2014; Wang, Chen, et al., 2020). Using this technique, it is possible to acquire extensive chemical data from the specimen with remarkable sensitivity (Wang, Rogers, et al., 2019). Therefore, the main aim of this research was to systematically analyze the modifications in volatile compounds throughout the processing of CER tea by employing HSPME and GC–MS techniques. The primary objective was to establish a theoretical foundation for enhancing the processing technology of CER tea.

## 2 MATERIALS AND METHODS

### 2.1 Samples from the CER tea manufacturing process

Fuyun 6 tea shoots with two leaves and one bud were picked and processed the same day at Pingyuan Yuanshanhu Agricultural Development Co., Ltd. located in Pingyuan city, Guangdong province, China.

**FIGURE 1:**
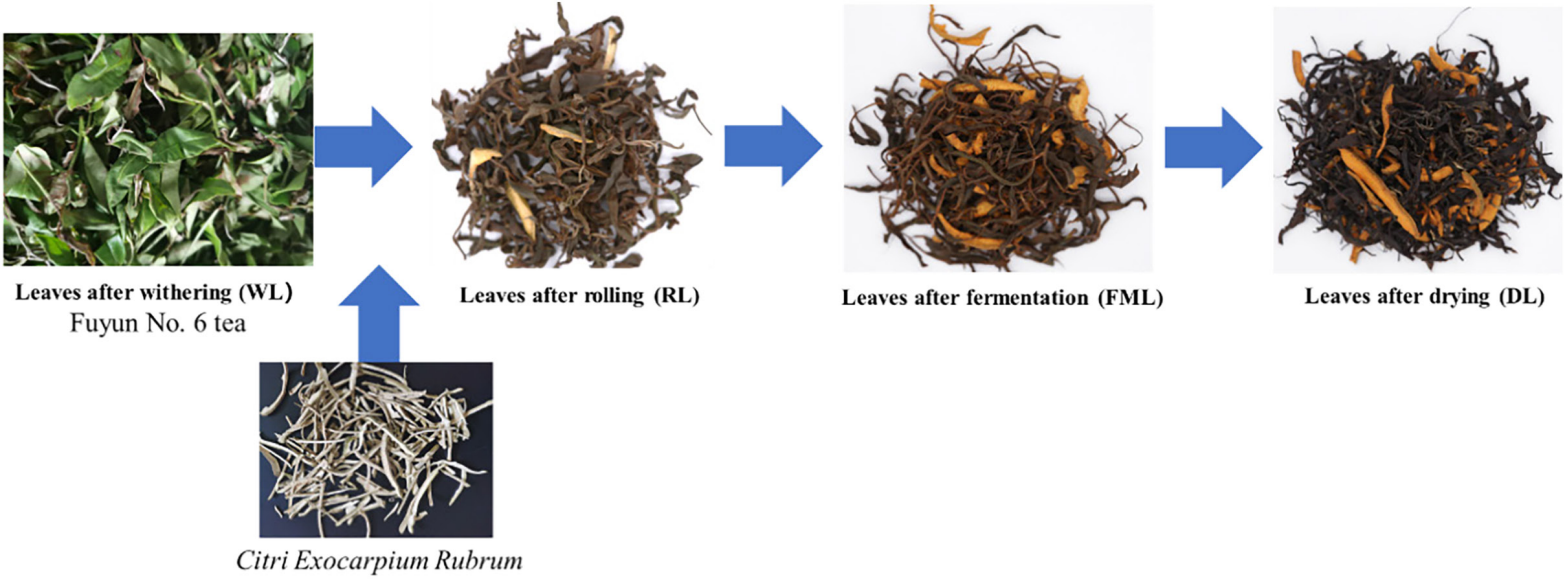
Diagram of samples during the manufacturing process.

Therewith, fresh leaves were processed as described by the national standard GB/T 35810–2018 published by the General Administration of Quality Supervision, Inspection and Quarantine of the People's Republic of China. The detailed processing process is as follows: the leaves, freshly harvested, were distributed inside a controlled environment where they were exposed for a duration of 16–20 h. The temperature was maintained at 25°C while the humidity level remained constant at 75%. When the leaves became soft and slightly fragrant, their moisture dropped to approximately 70%. After withering, the leaves were rolled for 1 h and 20 min to form a beautiful shape and aroma. During the rolling process, CER was added. The ratio of CER to withered leaves is 1:12. Then, the mixture was fermented for 2 h and finally dried, and the water content was less than 6% (Figure 1). To explore the changes in aroma resulting from biological transformations and heat variations during the primary processing stages of CER tea, we collected samples immediately after the completion of four crucial procedures: withered leaves (WL), rolled leaves (RL), fermented leaves (FML), and dried leaves (DL). To ensure accuracy and minimize errors, in the analysis of aroma composition, every sample was individually placed in liquid nitrogen for immediate freezing prior to being stored at −80°C. To maintain consistency, three biological replicates were performed within each group.

### 2.2 Sample extraction procedure

Powdered samples were prepared by grinding in liquid nitrogen. Each sample was weighed to 1 g and transferred to headspace vials (Agilent, Palo Alto, CA, USA). Subsequently, a saturated solution of NaCl and 10 μL of an internal standard solution with a concentration of 50 μg/mL were added. The vials were tightly sealed using crimp‐top caps equipped with TFE‐silicone headspace septa (Agilent). To extract the samples, the HS‐SPME method was employed.

During SPME analysis, the sample's headspace vial was placed into the extraction tip containing a 120 μm DVB/CAR/ PDMS extraction tip. This process occurred under consistent temperature conditions of 100°C for a duration of 5 min. Following the headspace extraction lasting 15 min, the solution underwent resolution at 250°C for an additional 5 min before undergoing GC–MS analysis.

### 2.3 GC–MS analysis

For GC–MS analysis, an 8890‐5977B system (Agilent J&W Scientific, Folsom, CA, USA) equipped with a DB‐5MS (5% phenyl‐polymethylsiloxane) capillary column (30 m × 0.25 mm × 0.25 μm) was used in the splitless injection mode with a high‐purity helium (99.999%) flow at 1.2 mL/min. The injector temperature was kept at 250°C and the detector at 280°C. The GC oven temperature was programmed from 40°C (3.5 min), increasing at 10°C/min to 100°C, at 7°C/min to 180°C, at 25°C/min to 280°C, and held for 5 min. Mass spectra were recorded in electron impact (EI) ionization mode at 70 eV. The quadrupole mass detector, ion source, and transfer line temperatures were set, respectively, at 150, 230, and 280°C. Mass spectra were scanned in the range m/z 50–500 amu at 1‐s intervals. Identification of volatile compounds was achieved by comparing the mass spectra with the data system library and linear retention index (Zhang et al., 2019).

### 2.4 Statistical analysis

The statistical function prcomp of SIMCA 14.0 was utilized to conduct principal component analysis (PCA). Heatmaps with dendrograms were used to present the results of the hierarchical cluster analysis (HCA) conducted on the data of volatile components. The R package Complex Heatmap was used to perform both hierarchical clustering analysis (HCA) and calculation of the Pearson correlation coefficient (PCC) between samples, using the cor function. For HCA, the normalized signal intensity (unit variance scaling) of metabolites is visualized as chromatography. After standardization and centralization, we conducted a K‐means cluster analysis to examine the variation in metabolite content across various samples.

We identified the metabolites that exhibited substantial regulation across different groups using a criterion of VIP≥1 and an absolute log_2_FC (fold change) ≥1. The OPLS‐DA result provides the extracted VIP value, along with a score graph and Pareto graph created using the R‐package MetaboAnalystR. Before conducting OPLS‐DA, the data underwent log transformation using a base of 2 and then was calculated for the mean median. To prevent overfitting, we conducted an arrangement test, comprising 200 arrangements.

The metabolites identified in the study were annotated through the utilization of the KEGG compound database (http://www.kegg.jp/kegg/compound/). Afterward, the annotated metabolites were integrated into the KEGG pathway database (http://www.kegg.jp/kegg/pathway.html) for mapping purposes. Subsequently, the mapped pathways containing metabolites exhibiting significant regulation were subjected to MSEA (metabolite sets enrichment analysis).

## 3 RESULTS AND DISCUSSION

### 3.1 Qualitative analysis of volatile components in CER tea processing

A total of 282 volatile compounds were identified from the samples of various processing methods of CER tea using the GC–MS detection platform. According to the chemical structure analysis, the volatile components mainly contained 80 terpenes, 53 esters, 39 heterocyclic compounds, 21 hydrocarbons, 18 ketones, 15 aromatic hydrocarbons, 13 alcohols, 11 aldehydes, 7 amines, 7 halogenated hydrocarbons, 6 phenols, 5 nitrogen‐containing compounds, 3 sulfur‐containing compounds, 2 acids, 1 ether, and 1 other compound (2,2′‐Bi1,3‐dioxolane). Terpene and ester volatile components were compounds with relatively rich species in CER tea, which laid the foundation for the formation of the aroma quality of CER tea.

**FIGURE 2:**
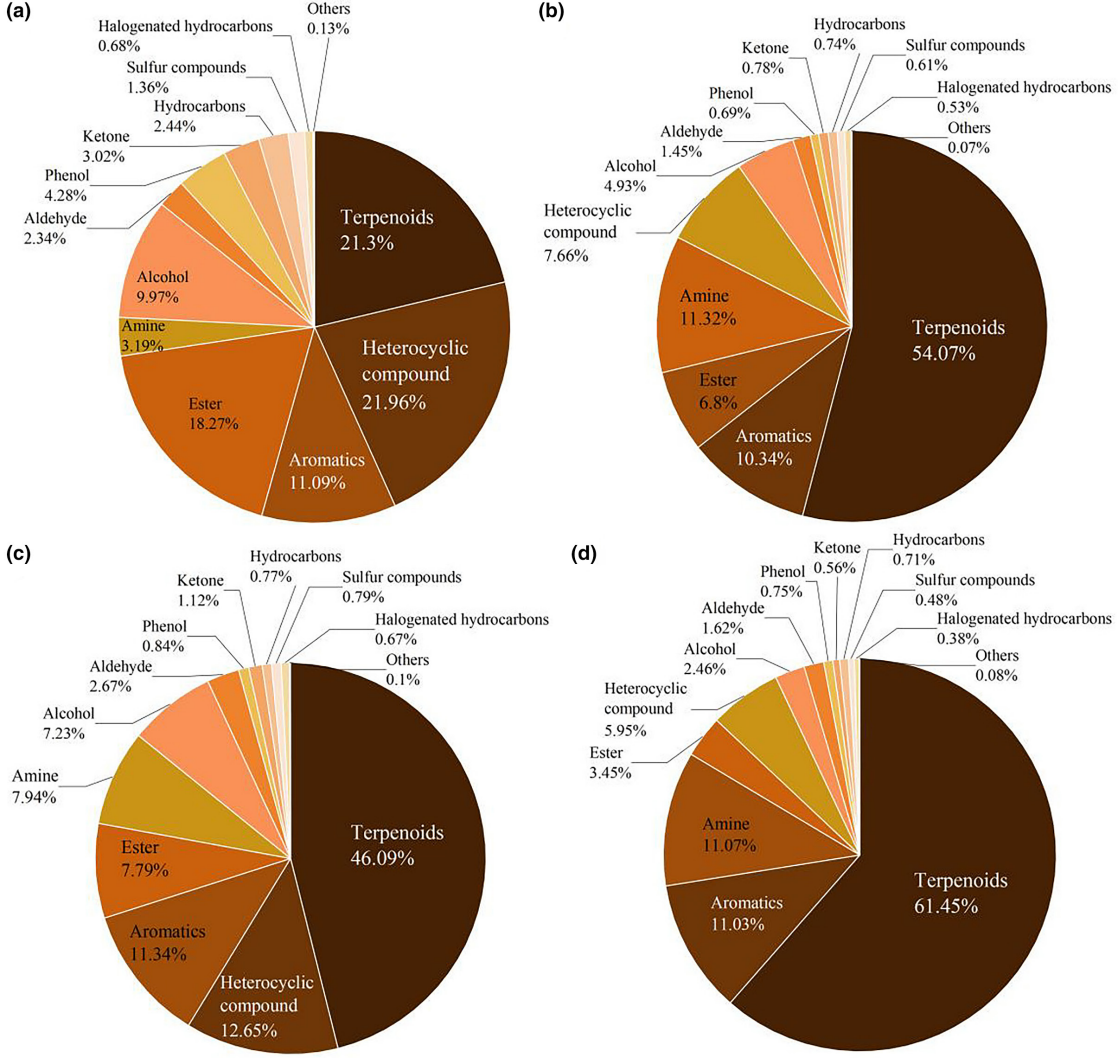
The ratios (%) of volatile compounds in four processing stages of CER tea. (a) WL, (b) RL, (c) FML, (d) DL.

The distribution of volatile compounds during CER tea processing is shown in Figure 2. The ratio (%) of terpenoids was the highest (WL, 21.30%; RL, 54.07%; FML, 46.09%; DL, 61.45%). Heterocyclic compound was the second abundant compound classification, but the content of each stage was significantly different (WL, 21.96%; RL, 7.66%; FML, 12.65%; DL, 5.95%). In detail, limonene was definitely the highest component (WL, 0.42%; RL, 19.38%; FML, 14.47%; DL, 22.37%), followed by γ‐terpinene (WL, 0.15%; RL, 9.60%; FML, 8.41%; DL, 14.27%). Linalool is considered to be the most important odorant in black tea (Yu et al., 2018), and it was also found in the processing of CER tea, with a ratio of 0.34%–4.19%.

Aroma is one of the important determinants of tea quality. It is worth noting that compared with traditional pure tea, the flavor of blend tea is more complex, which is due to the superposition and interaction effect of odors between tea and plant materials (Li et al., 2018; Wang, Zhu, et al., 2020). CER tea is a blend tea product made from CER and tea leaves. Compared with previous reports on citrus and tea (Wang, Zhu, et al., 2020), the content of terpene in our results is much higher. Research has indicated that the variety of degradation byproducts derived from terpene derivatives was extensive, primarily consisting of olefins and alcohols. These substances, notably β‐ocimene, γ‐terpinene, and longifolene, constituted the distinct aromatic elements found in citrus fruits like oranges and grapefruits (Cui et al., 2016) (Bazemore et al., 2003). The main volatile compounds that we can find during CER processing, such as D‐limonene and γ‐terpinene, are present in very low amounts during the wilting stage, but increase significantly during rolling, fermentation, and drying. Therefore, we supposed that D‐limonene and γ‐terpinene may mainly come from CER. The relatively high content of D‐limonene and γ‐terpinene in CER tea may be due to the simple addition effect between the volatile compounds of pure tea and CER. In addition, linalool was also detected in this study. Linalool is considered to be the key aroma component in most black tea, presenting floral or fruity aroma. Linalool was also detected in CER (Fan et al., 2024). Qi et al. (2020) found that linalool contributed the most to the aroma composition of citrus tea, and it had a fragrant floral scent. Linalool could be detected in the processing of CER tea in this study, but the main source is difficult to determine, because it is a key volatile compound in both black tea and CER.

### 3.2 Chemometric analysis and identification of different volatile compounds

To analyze the reliability and general features of these samples, the initial analysis involved the utilization of PCC, PCA, and HCA.

**FIGURE 3:**
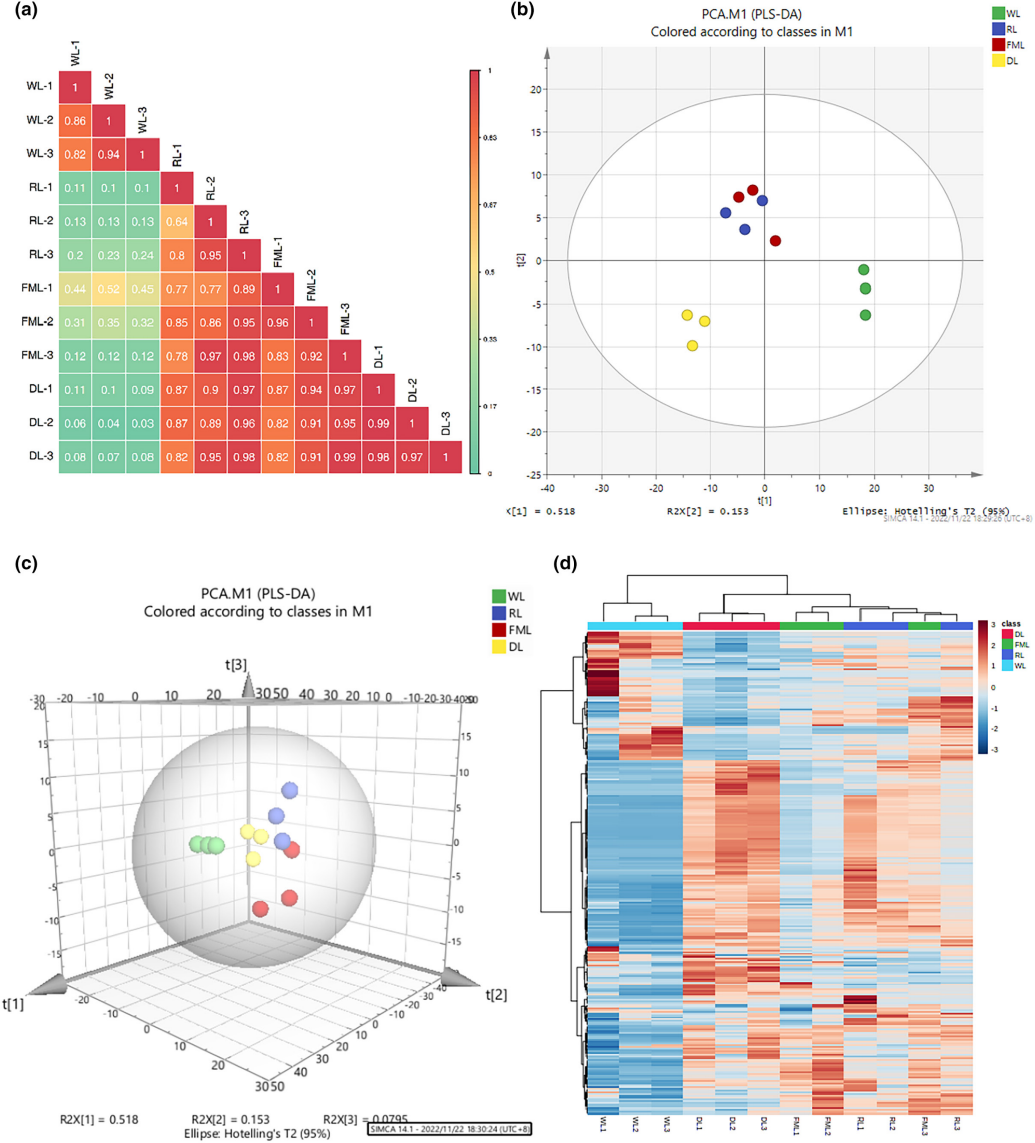
Identification of volatile compounds. (a) Correlation analysis using Pearson's correlation coefficient (PCC), (b) principal component analysis (PCA) of these four groups, (c) 3D plots of these four PCAs, (d) Hierarchical cluster analysis (HCA) of the identified compounds in different groups.

To evaluate biological repeatability between samples and groups, we used PCC for correlation analysis (Figure 3a). Through the calculation of the r^2^ value, the repeatability of the samples from each group was quite good. However, we found that the correlation coefficient between the withering sample and the dried sample was the smallest among the sample groups in different processing procedures of CER tea, indicating that the difference in quality components contained in the samples was the most significant, followed by the rolling sample and the fermentation sample. The correlation coefficients of samples in the rolling and fermentation stages are between 0.75 and 0.97, and those in the fermentation and drying stages are between 0.81 and 0.98. In summary, during processing, the aroma components of tea samples changed to some extent.

To preliminarily comprehend the overall discrepancies in metabolism among samples within each group and ascertain the level of variation within the group, we conducted principal component analysis through the utilization of SIMCA 14.1.0 software. The principal component scores are shown in Figure 3b,c. For the PCA plot, a greater distance between samples in the abscissa indicates a greater difference between metabolites. In the PCA 3D map, the first three principal components extracted, PC1, PC2, and PC3, are 51.80%, 15.30%, and 7.95%, respectively. The total cumulative contribution of PC1–PC3 is 75.05%. We could see from the two‐dimensional diagram of PCA that the tea samples in the key processing procedures of CER tea were distributed in three quadrants with regional distribution. The samples in the rolling stage and the fermentation stage were in the same area and interacted with each other, indicating that the differences in volatile components between the rolling stage and the fermentation stage were small.

In addition, HCA is used to extract information about the differences between the critical steps of the processing process based on the PCA model (Figure 3d). The four groups of samples are divided into three categories: one group was the withering samples, the other group was the rolling and fermentation samples, and the third group was the drying samples. The volatile component content changes with the progress of processing, and the volatile components in the key processing steps have a greater impact. It was possible that the CER peel was added during the rolling process, and the cells were crushed under the pressure of rolling. Intracellular enzymes participated in the oxidation process and accelerated metabolite transformation. In the rolling stage and the fermentation stage, there were rich metabolite transformations in the samples. Scientific investigations have established that fermentation plays a vital role in shaping the aromatic properties of black tea. The process of fermentation leads to a gradual transformation of the tea's aroma, transitioning from a robustly herbaceous taste to a delightful hint of sweetness, accompanied by floral or fruity undertones (Sharma et al., 2015). Therefore, it is speculated that rolling and fermentation are critical turning points in the processing of CER tea and should be strictly controlled.

To determine the different volatile components of CER tea in different processing stages, according to the PCA and HCA results, the OPLS‐DA model of adjacent processing stages was compared pairwise, and the amounts of different metabolites in adjacent stages were calculated (Table 1). A total of 140 compounds were screened in the withering and rolling stages, among which 137 components increased, mainly terpenes, heterocyclic compounds, and esters, which indicated that during rolling, polyphenols were decomposed by enzymatic oxidation and coupling reactions, and aromatic substances were gradually released. For example, terpene glycoside conjugates were hydrolyzed by β‐glucosidase or β‐primrose glucosidase to release free monoterpenes and sesquiterpenes, which led to an increase in terpene content. Furthermore, terpenoids have a low odor threshold and aromatic odor, which makes tea aroma change significantly (Hu et al., 2018), which shows that terpenoids play a key role in CER tea.

**TABLE 1:** The number of metabolites in different stages.

Group name	All sig diff	Downregulated	Upregulated
WL_vs_RL	140	3	137
RL_vs_FML	8	7	1
FML_vs_DL	74	28	46

Eight volatile components were screened in the rolling and fermentation stages, and the different metabolites were the least. This is consistent with the results of HCA, which aggregates the samples in rolling and fermentation stages, indicating that there is no obvious difference. Compared with the two stages, except vitispirane, the other seven volatile components showed a downward trend, including esters (isobutyl 2‐(4‐methylcyclohex‐3‐enyl)propan‐2‐yl carbonate), (3‐hexen‐1‐ol, acetate, (E)‐); ketones (1‐(2,3,4,5‐tetramethylphenyl)ethanone); hydrocarbons (2‐methyl‐7‐exo‐vinylbicyclo [4.2.0] oct‐1 (2)‐ene); terpenes (β‐phellandrene, bicyclo[3.1.1]heptane, 6,6‐dimethyl‐2‐methylene‐, (1S)‐, and camphene). There was no obvious change in volatile components in the rolling and fermentation stages.

A total of 74 differential volatile components were screened out in the fermentation and drying stages, of which 46 were ascending components, and terpenes were the main ones, and the substances mainly including sweet‐scented, floral‐or fruit‐scented decanal, γ‐juniperidine, β‐damascenone, (−)‐carvone, D‐Limonene, and γ‐terpinene were increasing. There were 28 reducing components, which were mainly ester compounds. Substances such as 2‐hexen‐1‐ol, (Z)‐, 3‐hexen‐1‐ol, (E)‐ of green grass flavor and acetic acid, decyl ester, 2‐butenoic acid, ethyl ester, (E)‐, and methyl salicylate of wintergreen oil flavor were decreased.

Therefore, the drying stage might be the key stage for the formation of the characteristic aroma of CER tea, while substances such as decanal, γ‐juniperidine, β‐damascenone, (−)‐carvone, D‐limonene, and γ‐terpinene, which have flower and fruit aroma properties, were highly correlated with the formation of the characteristic aroma of CER tea.

### 3.3 Changes in critical processes for volatile compounds during processing

**FIGURE 4:**
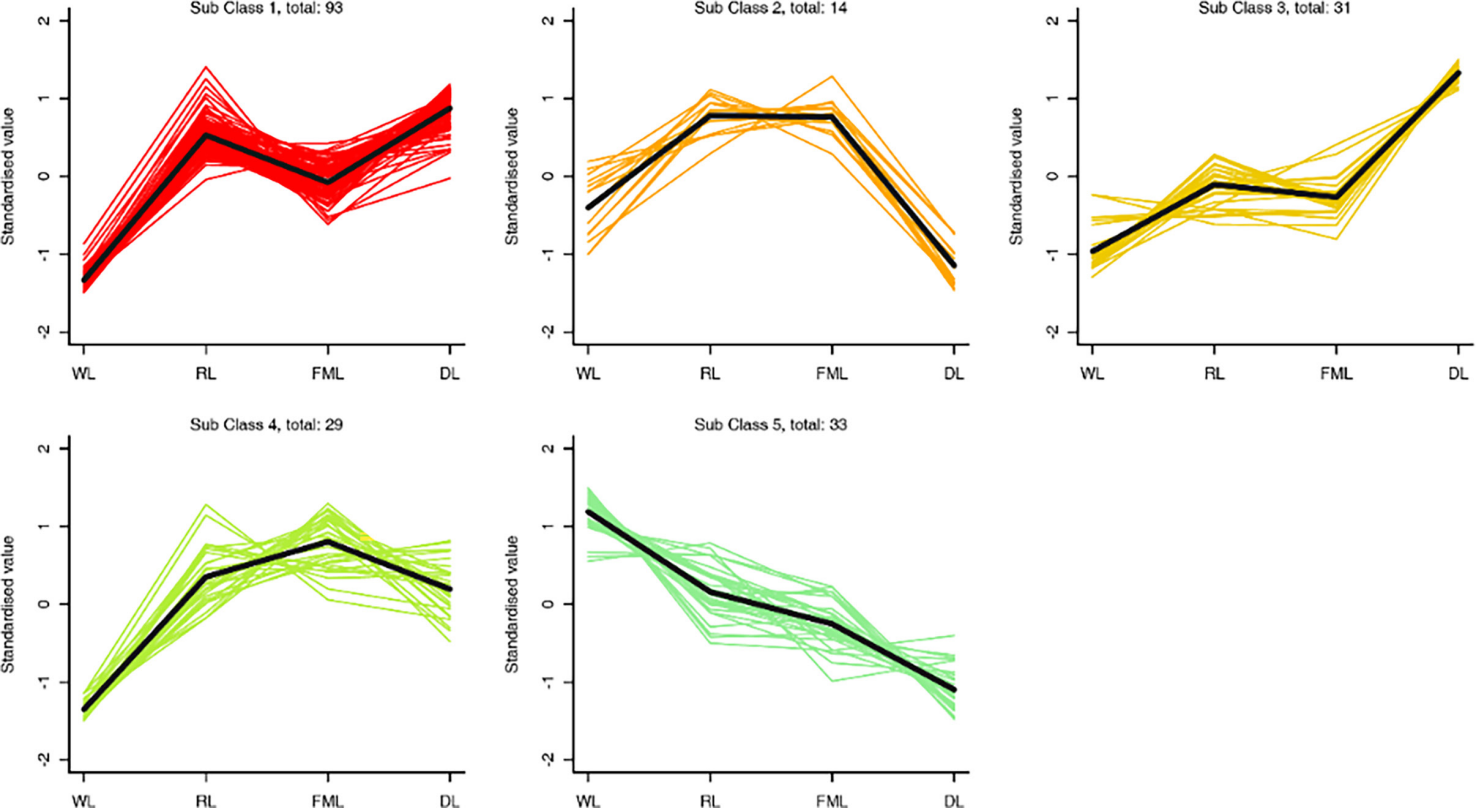
K indicates clustering analysis of differential metabolites.

To investigate the shifting pattern of metabolites throughout crucial procedures, the proportions of 200 distinct metabolites recognized based on the screening principles in the fading, will be examined, rolling, fermentation, and drying group comparisons were subjected to K‐means clustering analysis, which was divided into five subclasses (Figure 4).

K‐means clustering analysis showed that 124 metabolites of subclasses 1 and 3 were significantly increased in the rolling stage and drying stage. These volatile compounds may be closely related to the aroma of CER tea. Among them, benzeneacet aldehyde, dodecanal, trans‐nerolidol (1,6,10‐dodecatrien‐3‐OL, 3,7,11‐trimethyl‐,(E)‐), trans‐β‐ionone and β‐damascenone have floral and fruity odors, contributing to the fragrance of CER tea. The above odor molecules were also detected in other black teas. Benzene acetaldehyde has a rose odor and floral odor. The aroma intensity was highest in Assam black tea, and the relative content was relatively high in the Qimen black tea sample. In addition, Kang et al. (2019) also believe that phenylacetaldehyde may contribute most to the aroma formation of Ceylon black tea because its odor activity value (OAV) (514.68) was much higher than that of other teas (Kang et al., 2019). Phenylacetaldehyde was one of the key characteristic aroma components in black tea and one of the main floral sources in black tea. Trans‐β‐ionone has the fragrance of flowers, roses, and fruits, and it is found that the aroma concentration in Keemun black tea is the strongest. β‐damascenone represents the black tea (Jinjun Mei, Keemun, and Dianhong) OAVs in China and shows a high aroma contribution (Niu et al., 2022). Nerolidol is the main aroma component of Fujian oolong tea varieties, with floral and fruity characteristics (Zhu et al., 2015). Additionally, among the differential metabolites, we found gamma‐juniperidine and limonene. In the study conducted by Xiao et al. (2017), the authors employed a combination of GC‐O and OAV values, along with recombination and reduction techniques, to identify the crucial fragrance—contributing compounds in Ponkan orange. The results revealed that nonanal, hexanal, linalool, and limonene played a pivotal role in presenting the fragrance characteristics of this citrus fruit. γ‐Juniperidine has a citrus flavor, and limonene has a pleasant fruity flavor and is a key aroma component in citrus plants. We speculated that it was mainly derived from CER, which might be the key aroma component of CER tea that is different from other black teas.

K‐means clustering analysis also showed that 43 metabolites of subclasses 2 and 4 presented a significant increase in the rolling stage and a significant decrease in the drying stage. No significant changes were observed in the rolling and fermentation stages. Among them, β‐ionone, 2‐hexen‐1‐ol, (Z)‐, 3‐hexen‐1‐ol, (E)‐, (E)‐linalool oxide (furanoid), and methyl salicylate were also detected. The 33 metabolites of the five subtypes showed a significant downward trend in the rolling, fermentation, and drying stages. Among them, linalool and geraniol were the differential metabolites of this subclass. Linalool and linalool oxide (furanoid) had sweet, fruity, and banksia rose flavors, and geraniol was floral (threshold in water was 7.5 μg/kg) (Rychlik et al., 1998). 2‐hexen‐1‐ol, (Z)‐ intense green leaf and fruit aroma, and 3‐hexen‐1‐ol, (E)‐ intense fresh green leaf aroma. Linalool has been found to be one of the key aroma‐forming components of black tea (Joshi & Gulati, 2015). The Qimen black tea contained cis/trans‐linalool oxide (furan) and erythritol with rich fragrance; the trans‐linalool oxide in Yunnan Gongfu; geraniol in Tanyang Gongfu, Yunnan Gongfu and Qimen Gongfu black tea all had the highest fragrance dilution (flavor dilution, FD), which was the key fragrance‐presenting substance (Xiao et al., 2017). In Darjeeling black teas, (Z)‐3‐hexene‐1 alcohol, acetate, linalool, hexanal, and limonene (especially linalool with extremely high OAV (2468.98)) are considered to be key odorants contributing to the typical fruit aroma of Darjeeling black teas (Kang et al., 2019). KASUGA et al. (Kasuga et al., 2010) reported that linalool, 4‐ hydroxy ‐2,5‐ dimethyl ‐3(H)‐ furanone, geraniol, coumarin, (E)‐2‐ hexenoic acid, 3‐ methylbutyric acid, and phenylacetic acid are key aroma components of five different brands of Sri Lankan black tea. From the above studies, we find that the key aroma components of black tea obtained by analysis in different studies also vary significantly, which may be related to the different raw materials of the samples selected, the differences in the processing technology of the samples, and the differences in the methods used for aroma extraction.

In this study, the linalool content decreased during the rolling and fermentation stages, and the trans‐linalool oxide (furanoid) content increased, while both exhibited a downward trend during the drying stage. The possible reason was that during the fermentation stage, the linalool content was decreased due to hydrolysis under the action of oxidase. This was basically consistent with the variation law of linalool content during the fermentation of the “Yinghong No 9” black tea variety (Yang et al., 2016).

As previously stated, extensive research has been conducted on the primary aromas present in certain variants of black tea. It is noteworthy that our investigation aligns with the findings reported in earlier literature regarding these characteristic scents. For example, phenylacetaldehyde, linalool, geraniol, and trans beta‐ion compounds. Therefore, based on the results of the GC–MS method and statistical analysis in this study, as well as the summary results of published literature, the common and determined odor of CER tea can be predicted, including phenylacetaldehyde, linalool, geraniol, and trans β‐ionone and β‐damascenone. Compared with the aroma components of other black teas, γ‐juniperidine and D‐limonene were also detected in this paper, which were speculated to be mainly from CER, and might be the key characteristic aroma components of CER tea different from other black teas, which would provide a theoretical reference for the research of CER tea.

### 3.4 Metabolic pathways of aroma component changes during processing

**FIGURE 5:**
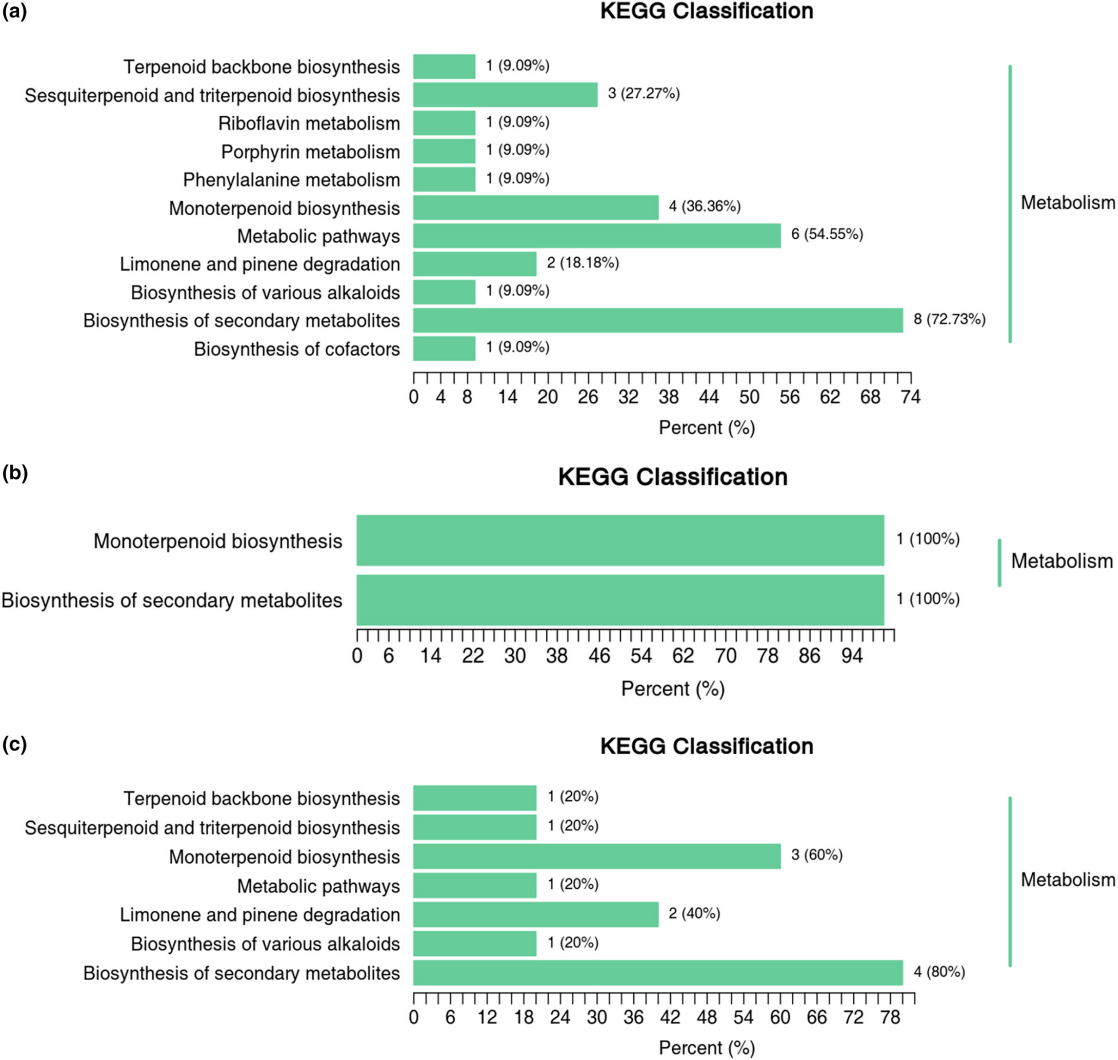
Metabolic pathway of aroma components in CER tea processing. (a) WL_vs_RL, (b) RL_vs_FML, (c) FML_vs_DL.

In this investigation, we utilized the KEGG database to annotate and present the differential metabolites. As shown in Figure 5, 11 pathways are enriched in WL versus RL comparison; two pathways are enriched in RL versus FML comparison; seven pathways are enriched in FML versus DL comparison. The common enrichment pathways during tea processing were monoterpene biosynthesis and biosynthesis of secondary metabolites, indicating that the differences in the relative content of some volatile metabolites in CER tea processing may be caused by the monoterpene biosynthesis reaction. Monoterpene biosynthesis is an important regulatory pathway affecting the aroma of tea. The relative content of some volatile metabolites may be regulated by monoterpene biosynthesis, thereby changing the original aroma quality of black tea. From the metabolic pathway analysis in this study, we identified terpineol and limonene as the key monoterpenoid differential metabolites. Terpineol has been identified in a variety of tea leaves and is thought to contribute to floral aroma formation (Chigo‐Hernandez & Tomasino, 2023). In this study, the content of α‐terpineol was low in the wilting stage but significantly increased in the fermentation stage, which may be one of the reasons why CER tea has rich aroma and various aroma characteristics in different processing stages. Limonene has a lemon‐like odor, indicating that the addition of CER during processing can increase the formation of fruity aroma in tea products (Ademosun et al., 2018; Mehl et al., 2014). Moreover, limonene, also many single terpenoid material synthesis intermediates, has an important influence on the formation of single terpenoid substances. In addition, biosynthesis of secondary metabolic pathways was also enrichment pathways common to processing. Many aroma compounds in tea were produced and degraded by secondary metabolic biosynthetic pathways. Terpineol and limonene also belong to the secondary metabolism synthesis pathway of related compounds. In summary, monoterpenoid biosynthesis pathway and secondary metabolism pathway may be the key metabolic pathways leading to the changes in the relative content of metabolites during CER tea processing. The results of the present study provide a novel theoretical basis for the development of CER tea.

## 4 CONCLUSION

In this study, the main aroma components of CER tea were determined by HS‐SPME/GC–MS, and the changes in volatile compounds in different preparation stages were analyzed. With the processing of CER tea, the volatile content changes significantly, and the aroma properties at different processing stages are also different. The differential metabolites of terpenes and esters are abundant in the processing. Most of the terpenoid differential metabolites increased in the rolling stage, decreased in the fermentation stage, and increased in the drying stage. Terpenes have a low odor threshold and aromatic odor and may be the key volatile substances of CER tea. Compared with other black tea aroma components, limonene were also detected in this paper, which was mainly fruity odor. It is speculated that they mainly came from CER, which may be the key characteristic aroma components that distinguish CER tea from other black teas. KEGG analysis also found that monoterpenes may be important contributors to the aroma of black tea. This paper could provide a theoretical reference for processing technology research on CER tea.

## AUTHOR CONTRIBUTIONS

**Qiaoyi Zhou:** Data curation (equal); funding acquisition (equal); writing – original draft (equal); writing – review and editing (equal). **Dongxia Liang:** Writing – original draft (equal); writing – review and editing (equal). **Caijin Ling:** Funding acquisition (equal); methodology (equal); resources (equal). **Liyang Gao:** Investigation (equal). **Zhi Ling:** Investigation (equal).

## FUNDING INFORMATION

This study was funded by Modern Agricultural Industry Technology System Innovation Team Construction Project of Guangdong Province with Agricultural Products as Unit (Tea) (Project Code: 2024KJ120); The Innovation Fund projects of Guangdong Provincial Key Laboratory of Tea Plant Resources Innovation and Utilization (Project Code: 2021CX03); Zijin County Science and Technology plan project (Project Code: 202202); Heyuan City Science and Technology plan project (Project Code: Heke 2021030).

## CONFLICT OF INTEREST STATEMENT

The authors declare no conflict of interest.

## Data Availability

The data that support the findings of this study are available on request from the corresponding author. The data are not publicly available due to privacy or ethical restrictions.
